# Epidemiological and clinical characteristics of central nervous system infections in a tertiary center: A retrospective study

**DOI:** 10.1002/hsr2.1099

**Published:** 2023-02-06

**Authors:** Bikram P. Gajurel, Subarna Giri, Shivani Rayamajhi, Niharika Khanal, Sagar Bishowkarma, Aman Mishra, Ragesh Karn, Reema Rajbhandari, Rajeev Ojha

**Affiliations:** ^1^ Department of Neurology Tribhuvan University Teaching Hospital Kathmandu Nepal; ^2^ Maharajgunj Medical Campus, Tribhuvan University Institute of Medicine Kathmandu Nepal

**Keywords:** CNS infection, encephalitis, meningitis, meningoencephalitis

## Abstract

**Background and Aims:**

Central nervous system (CNS) infection is one of the most common causes of morbidity, mortality, and hospital admission worldwide. The natural history of CNS infection is quite fatal. Early diagnosis and treatment have been proven to have a crucial role in patients' survival. The aim of this study was to identify the epidemiological and clinical patterns of patients diagnosed with CNS infections.

**Methods:**

This study is a retrospective study conducted in a tertiary level hospital in Nepal in which patient diagnosed with CNS infections (September 2019 to 2021) were included. Data were collected and analyzed in SPSS.

**Results:**

The mean age of the 95 patients included in the study was 45.18 ± 19.56. Meningoencephalitis (*n* = 44, 46.30%) was the most common infection diagnosed. Patients belonging to the age group 30−60 years had a higher frequency of focal neurological deficit, and other classical clinical features. All the patients who died during the treatment had associated comorbidities but no concurrent infections. Altered sensorium, fever, and headache were the common presenting symptoms in all the recovered patients.

**Conclusion:**

To ensure optimum disease outcome, early diagnosis and prompt management are crucial. For this, recognizing the local disease patterns in terms of disease distribution, commonly implicated aetiologies, presenting symptoms, and prognostic factors is of utmost importance.

## 1 INTRODUCTION

Central nervous system (CNS) infection is one of the most common causes of morbidity, mortality, and hospital admission worldwide. Meningitis, encephalitis, meningoencephalitis, and brain abscess are common presentations of CNS infections^1^, ^2^, ^3^, ^4^ which are caused by a range of organisms including bacteria, viruses, fungi, parasites, and prions.^1^

CNS infection clinically manifests as fever, headache, and vomiting associated with seizure, loss of consciousness, altered sensorium, focal neurologic deficit, and blurring of vision. However, clinical manifestation varies according to different etiology and brain tissue involvement. The natural history of CNS infection is quite fatal. Early diagnosis and treatment have been proven to have a crucial role in patients' survival.^1^

It is usually diagnosed through the clinical presentation, blood tests, blood culture, and electroencephalography and confirmed by cerebrospinal fluid (CSF) analysis and neuroimaging in low‐income countries like Nepal. Other invasive tests like polymerase chain reaction and biopsy are usually not done. However, there is no consistent result regarding diagnostics methods. Lumbar puncture for CSF analysis is almost always done in patients with high clinical suspicion of CNS infection. Electroencephalogram (EEG) is usually done if meningoencephalitis or encephalitis is suspected with manifestations like seizure and altered sensorium.

There has been significant geographical variation in distribution, clinical presentation, and outcome. One of the few studies from Nepal has shown Japanese Encephalitis (JE) and enterovirus as the most common cause of CNS infection in adults while *S. pneumoniae* and *N. meningitidis* still stand as the most common bacterial etiology causing CNS infection, tubercular meningitis is also the common diagnosis.^3^ Results of different studies from different parts of the world do not correlate with each other and vary significantly.^5^, ^6^

Despite the high prevalence and burden in developing countries like Nepal and other South Asian countries, there are limited articles on epidemiological aspects of CNS infection. Thus, a retrospective study to observe the various epidemiological characteristics of a CNS infection patient was conducted in one of the largest tertiary centers in Nepal.

## 2 METHODS

### 2.1 Study design

The study was conducted in a retrospective design, in Tribhuvan University Teaching Hospital. Our institution is a major referral and tertiary level hospital in Nepal with high patient flow.

### 2.2 Inclusion criteria

Patients with diagnosis of meningitis, meningoencephalitis, encephalitis, and parasitic infection of the brain and under the care of the neurology department of Tribhuvan University Teaching Hospital from September 1, 2019 to September 1, 2021 were included in the study.

### 2.3 Exclusion criteria

All those patients whose diagnosis was uncertain and inconclusive were excluded from the study. Patients with incomplete or missing data were also excluded from the study.

### 2.4 Sampling

A non‐probability, convenience sampling was done for the sample size selection of our study. We reviewed the charts of the hospital's neurology department from September 2019 to 2021. The data were collected based on a pre designed proforma sheet manually. The data were categorized into the domains of demography, clinical presentation, investigation, diagnosis, treatment, and outcome. The first set of data included age, sex, address (based on provincial division of country), marital status, comorbidities, concurrent infections, previous treatment/surgery history, and placement of invasive devices. The second set of data included clinical features (fever, vomiting, seizures, headache, altered mental status, focal neurologic deficit, and others), signs (neck stiffness, Brudzinski sign, Kernig sign, papilledema), investigations (lumbar puncture, imaging), and outcomes of the patient.

### 2.5 Statistical analysis

The raw data were entered and maintained in Microsoft Excel (Ver 2016). The analysis of the data was done using SPSS 21 (IBM Corp. Released 2012. IBM SPSS Statistics for Windows; Version 21.0; IBM Corp.). Initially, a descriptive analysis of the data was done. Categorical data was presented in terms of frequency and percentage. Continuous data were presented as mean and median. A *p*‐value of 0.05 or less has been considered statistically significant.

### 2.6 Ethical consideration

Ethical clearance was obtained from the Institutional Review Board of our institution, before commencing with the data collection (Reference Number: 188 [6‐11] E2/078/079). There were no human harm or direct contact with the patient. The data collection was done in such a manner that no identifying characteristics of the patient had been recorded in the excel sheets.

## 3 RESULT

After a retrospective review of the chart of patients with CNS infection admitted to our hospital (September 1, 2019 to September 1, 2021), a total of 120 patients were identified but only 95 patients were included in the study. Twenty‐five patients were excluded due to incomplete data. The mean age group of the patients was 45.18 ± 19.56. The mean duration of hospital stay was 13.97 ± 10.79 days. Meningoencephalitis (*n* = 44, *N* = 95, 46.30%) was the most common infection diagnosed. Majority of the patients didn't have positive Kernig (*n* = 73, *N* = 95, 76.80%) and Brudzinski sign (*n* = 80, *N* = 95, 84.20%). Lumbar puncture was done in 75.80% (*n* = 72, *N* = 95) of the total patient. The detailed characteristics of the study population are shown in Table 1. Age group‐wise comparison of demographic, etiological, and clinical characteristics is also tabulated in Table 1. Out of the total patient who died, 75% (*n* = 3, *N* = 4) was noted in the age group 60−90 years. Patients belonging to the age group 30−60 years had a higher frequency of focal neurological deficit, and other classical clinical features.

**Table 1:** Age‐wise detailed characteristics of study population (*n* = 95).

			All (*n* = 95)	Age (10−30) (*n* = 27)	Age (30−60) (*n* = 42)	Age (60−90) (*n* = 26)	Row total
Age			45.18 ± 19.56	22.11 ± 3.71	44.59 ± 9.68	70.07 ± 7.15	
Duration of hospital stay			13.97 ± 10.79	16.14 ± 12.59	14.11 ± 11.25	11.46 ± 7.33	
Sex	Female	*n*	39	17	13	9	39
		%	41.10	43.6	33.3	23.1	100.0
	Male	*n*	56	10	29	17	56
		%	58.90	17.9	51.8	30.4	100.0
Province number	1	*n*	2	2	0	0	2
		%	2.10	100.0	0.0	0.0	100.0
	2	*n*	13	4	8	1	13
		%	13.70	30.8	61.5	7.7	100.0
	3	*n*	46	13	17	16	46
		%	48.40	28.3	37.0	34.8	100.0
	4	*n*	24	4	12	8	24
		%	25.30	16.7	50.0	33.3	100.0
	5	*n*	7	3	3	1	7
		%	7.40	42.9	42.9	14.3	100.0
	7	*n*	3	1	2	0	3
		%	3.20	33.3	66.7	0.0	100.0
Etiology	Autoimmune	*n*	5	3	1	1	5
		%	5.30	60.0	20.0	20.0	100.0
	Bacterial	*n*	11	4	5	2	11
		%	11.60	36.4	45.5	18.2	100.0
	Fungal	*n*	7	1	4	2	7
		%	7.40	14.3	57.1	28.6	100.0
	Unknown	*n*	12	1	4	7	12
		%	12.60	8.3	33.3	58.3	100.0
	Tapeworm	*n*	3	1	2	0	3
		%	3.20	33.3	66.7	0.0	100.0
	Tubercular	*n*	31	12	13	6	31
		%	32.60	38.7	41.9	19.4	100.0
	Viral	*n*	26	5	13	8	26
		%	27.40	19.2	50.0	30.8	100.0
Diagnosis	Encephalitis	*n*	16	2	10	4	16
		%	16.80	12.5	62.5	25.0	100.0
	Meningitis	*n*	35	11	19	5	35
		%	36.80	31.4	54.3	14.3	100.0
	Meningoencephalitis	*n*	44	14	13	17	44
		%	46.30	31.8	29.5	38.6	100.0
Marital status	Married	*n*	78	14	38	26	78
		%	82.10	17.9	48.7	33.3	100.0
	Unmarried	*n*	17	13	4	0	17
		%	17.90	76.5	23.5	0.0	100.0
Comorbidities	Cardiac disease	*n*	15	0	7	8	15
		%	15.8	0.0	46.7	53.3	100.0
	Diabetes	*n*	9	1	4	4	9
		%	9.50	11.1	44.4	44.4	100.0
	None	*n*	51	24	20	7	51
		%	53.70	47.1	39.2	13.7	100.0
	Others	*n*	20	2	11	7	20
		%	21.10	10.0	55.0	35.0	100.0
Concurrent infections	Yes	*n*	21	7	10	4	21
		%	22.10	33.3	47.6	19.0	100.0
	No	*n*	74	20	32	22	74
		%	77.90	27.0	43.2	29.7	100.0
Previous treatment/referral	No	*n*	38	15	10	13	38
		%	40	39.5	26.3	34.2	100.0
	Yes	*n*	47	12	32	13	57
		%	60	21.1	56.1	22.8	100.0
History of surgery	No	*n*	85	24	37	24	85
		%	89.50	28.2	43.5	28.2	100.0
	Yes	*n*	10	3	5	2	10
		%	10.50	30.0	50.0	20.0	100.0
Invasive device placed	No	*n*	90	27	38	25	90
		%	94.70	30.0	42.2	27.8	100.0
	Yes	*n*	5	0	4	1	5
		%	5.30	0.0	80.0	20.0	100.0
Fever	No	*n*	31	8	9	14	31
		%	32.60	25.8	29.0	45.2	100.0
	Yes	*n*	64	19	33	12	64
		%	67.40	29.7	51.6	18.8	100.0
Headache	No	*n*	44	12	15	17	44
		%	46.30	27.3	34.1	38.6	100.0
	Yes	*n*	51	15	27	9	51
		%	53.70	29.4	52.9	17.6	100.0
Vomiting	No	*n*	61	13	24	24	61
		%	64.20	21.3	39.3	39.3	100.0
	Yes	*n*	34	14	18	2	34
		%	35.80	41.2	52.9	5.9	100.0
Altered sensorium	No	*n*	39	13	19	7	39
		%	41.10	33.3	48.7	17.9	100.0
	Yes	*n*	56	14	23	19	56
		%	58.90	25.0	41.1	33.9	100.0
Seizures	No	*n*	63	16	28	19	63
		%	66.30	25.4	44.4	30.2	100.0
	Yes	*n*	32	11	14	7	32
		%	33.70	34.4	43.8	21.9	100.0
Neck stiffness	No	*n*	60	15	29	16	60
		%	63.20	25.0	48.3	26.7	100.0
	Yes	*n*	35	12	13	10	35
		%	36.80	34.3	37.1	28.6	100.0
Kernig sign	No	*n*	73	19	35	19	73
		%	76.80	26.0	47.9	26.0	100.0
	Yes	*n*	22	8	7	7	22
		%	23.20	36.4	31.8	31.8	100.0
Brudzinski sign	No	*n*	80	22	37	21	80
		%	84.20	27.5	46.3	26.3	100.0
	Yes	*n*	15	5	5	5	15
		%	15.80	33.3	33.3	33.3	100.0
Papilledema	No	*n*	93	25	42	26	93
		%	97.90	26.9	45.2	28.0	100.0
	Yes	*n*	2	2	0	0	2
		%	2.10	100.0	0.0	0.0	100.0
Focal neurologic deficit	No	*n*	64	22	25	17	64
		%	67.40	34.4	39.1	26.6	100.0
	Yes	*n*	31	5	17	9	31
		%	32.60	16.1	54.8	29.0	100.0
Need for lumbar puncture	No	*n*	23	7	9	7	23
		%	24.20	30.4	39.1	30.4	100.0
	Yes	*n*	72	20	33	19	72
		%	75.80	27.8	45.8	26.4	100.0
Need for electroencephalogram	No	*n*	84	20	42	22	84
		%	88.40	23.8	50.0	26.2	100.0
	Yes	*n*	11	7	0	4	11
		%	11.60	63.6	0.0	36.4	100.0
MRI findings	Abnormal	*n*	32	9	16	7	32
		%	33.70	28.1	50.0	21.9	100.0
	Normal	*n*	15	5	6	4	15
		%	15.80	33.3	40.0	26.7	100.0
	Not done	*n*	48	13	20	15	48
		%	50.50	27.1	41.7	31.3	100.0
Outcome	Death	*n*	4	1	0	3	4
		%	4.20	25.0	0.0	75.0	100.0
	Improving	*n*	53	19	27	7	53
		%	55.80	35.8	50.9	13.2	100.0
	Resolved	*n*	38	7	15	16	38
		%	40	18.4	39.5	42.1	100.0

Fungal infection and CNS infection with unknown etiology was diagnosed in the elderly population (55.00 ± 17.39 and 56.17 ± 18.90, respectively) compared to other etiological groups. The episode of seizure (*n* = 12), need for lumbar puncture test (*n* = 24), and resolution of the infection during hospital stay (*n* = 14) were commonly noted in viral pathology. Etiology‐wise characteristics of the patients are shown in Table 2.

**Table 2:** Etiology‐wise detailed characteristics of the study population (*n* = 95).

			Autoimmune (*n* = 5)	Bacterial (*n* = 11)	Fungal (*n* = 7)	Tapeworm (*n* = 3)	Tubercular (*n* = 31)	Viral (*n* = 26)	Unknown (*n* = 12)	Row total
Age			35.20 ± 21.15	41.09 ± 22.16	55.00 ± 17.39	38.66 ± 15.53	39.22 ± 17.11	48.96 ± 19.85	56.17 ± 18.90	
Duration of hospital stay			19.60 ± 9.12	11.27 ± 6.13	14.28 ± 9.91	2.33 ± 1.52	18.41 ± 14.36	12.65 ± 7.69	8.16 ± 4.95	
Sex	Female	*n*	4	3	1	1	16	10	4	39
		%	10.3	7.7	2.6	2.6	41.0	25.6	10.3	100.0
	Male	*n*	1	8	6	2	15	16	8	56
		%	1.8	14.3	10.7	3.6	26.8	28.6	14.3	100.0
Province number	1	*n*	0	0	1	0	1	0	0	2
		%	0.0	0.0	50.0	0.0	50.0	0.0	0.0	100.0
	2	*n*	0	3	1	0	5	4	0	13
		%	0.0	23.1	7.7	0.0	38.5	30.8	0.0	100.0
	3	*n*	3	4	1	1	14	14	9	46
		%	6.5	8.7	2.2	2.2	30.4	30.4	19.6	100.0
	4	*n*	2	4	4	0	6	5	3	24
		%	8.3	16.7	16.7	0.0	25.0	20.8	12.5	100.0
	5	*n*	0	0	0	2	3	2	0	7
		%	0.0	0.0	0.0	28.6	42.9	28.6	0.0	100.0
	7	*n*	0	0	0	0	2	1	0	3
		%	0.0	0.0	0.0	0.0	66.7	33.3	0.0	100.0
Diagnosis	Encephalitis	*n*	1	1	0	3	0	10	1	16
		%	6.3	6.3	0.0	18.8	0.0	62.5	6.3	100.0
	Meningitis	*n*	2	2	6	0	19	3	3	35
		%	5.7	5.7	17.1	0.0	54.3	8.6	8.6	100.0
	Meningoencephalitis	*n*	2	8	1	0	12	13	8	44
		%	4.5	18.2	2.3	0.0	27.3	29.5	18.2	100.0
Marital status	Married	*n*	5	7	6	3	25	22	10	78
		%	6.4	9.0	7.7	3.8	32.1	28.2	12.8	100.0
	Unmarried	*n*	0	4	1	0	6	4	2	17
		%	0.0	23.5	5.9	0.0	35.3	23.5	11.8	100.0
Comorbidities	Cardiac disease	*n*	1	0	1	1	5	4	3	15
		%	6.7	0.0	6.7	6.7	33.3	26.7	20.0	100.0
	Diabetes	*n*	0	2	2	0	2	2	1	9
		%	0.0	22.2	22.2	0.0	22.2	22.2	11.1	100.0
	None	*n*	4	6	3	2	20	12	4	51
		%	7.8	11.8	5.9	3.9	39.2	23.5	7.8	100.0
	Others	*n*	0	3	1	0	4	8	4	20
		%	0.0	15.0	5.0	0.0	20.0	40.0	20.0	100.0
Concurrent infections	Yes	*n*	0	4	2	0	10	2	3	21
		%	0.0	19.0	9.5	0.0	47.6	9.5	14.3	100.0
	No	*n*	5	7	5	3	21	24	9	74
		%	6.8	9.5	6.8	4.1	28.4	32.4	12.2	100.0
Previous treatment/referral	No	*n*	3	5	4	2	10	9	5	38
		%	7.9	13.2	10.5	5.3	26.3	23.7	13.2	100.0
	Yes	*n*	2	6	3	1	21	17	7	57
		%	3.5	10.5	5.3	1.8	36.8	29.8	12.3	100.0
History of surgery	No	*n*	4	10	7	3	26	24	11	85
		%	4.7	11.8	8.2	3.5	30.6	28.2	12.9	100.0
	Yes	*n*	1	1	0	0	5	2	1	10
		%	10.0	10.0	0.0	0.0	50.0	20.0	10.0	100.0
Invasive device placed	No	*n*	5	11	6	3	29	26	10	90
		%	5.6	12.2	6.7	3.3	32.2	28.9	11.1	100.0
	Yes	n	0	0	1	0	2	0	2	5
		%	0.0	0.0	20.0	0.0	40.0	0.0	40.0	100.0
Fever	No	*n*	3	0	3	3	5	11	6	31
		%	9.7	0.0	9.7	9.7	16.1	35.5	19.4	100.0
	Yes	*n*	2	11	4	0	26	15	6	64
		%	3.1	17.2	6.3	0.0	40.6	23.4	9.4	100.0
Headache	No	*n*	3	6	2	1	11	14	7	44
		%	6.8	13.6	4.5	2.3	25.0	31.8	15.9	100.0
	Yes	*n*	2	5	5	2	20	12	5	51
		%	3.9	9.8	9.8	3.9	39.2	23.5	9.8	100.0
Vomiting	No	*n*	4	6	5	3	16	18	9	61
		%	6.6	9.8	8.2	4.9	26.2	29.5	14.8	100.0
	Yes	*n*	1	5	2	0	15	8	3	34
		%	2.9	14.7	5.9	0.0	44.1	23.5	8.8	100.0
Altered sensorium	No	*n*	1	3	5	3	18	5	4	39
		%	2.6	7.7	12.8	7.7	46.2	12.8	10.3	100.0
	Yes	*n*	4	8	2	0	13	21	8	56
		%	7.1	14.3	3.6	0.0	23.2	37.5	14.3	100.0
Seizures	No	*n*	3	6	5	1	25	14	9	63
		%	4.8	9.5	7.9	1.6	39.7	22.2	14.3	100.0
	Yes	*n*	2	5	2	2	6	12	3	32
		%	6.3	15.6	6.3	6.3	18.8	37.5	9.4	100.0
Neck stiffness	No	*n*	3	6	6	3	18	19	5	60
		%	5.0	10.0	10.0	5.0	30.0	31.7	8.3	100.0
	Yes	*n*	2	5	1	0	13	7	7	35
		%	5.7	14.3	2.9	0.0	37.1	20.0	20.0	100.0
Kernig sign	No	*n*	4	7	6	3	25	22	6	73
		%	5.5	9.6	8.2	4.1	34.2	30.1	8.2	100.0
	Yes	*n*	1	4	1	0	6	4	6	22
		%	4.5	18.2	4.5	0.0	27.3	18.2	27.3	100.0
Brudzinski sign	No	*n*	4	8	7	3	28	23	7	80
		%	5.0	10.0	8.8	3.8	35.0	28.8	8.8	100.0
	Yes	*n*	1	3	0	0	3	3	5	15
		%	6.7	20.0	0.0	0.0	20.0	20.0	33.3	100.0
Papilledema	No	*n*	4	11	7	3	30	26	12	93
		%	4.3	11.8	7.5	3.2	32.3	28.0	12.9	100.0
	Yes	*n*	1	0	0	0	1	0	0	2
		%	50.0	0.0	0.0	0.0	50.0	0.0	0.0	100.0
Focal neurologic deficit	No	*n*	2	8	6	2	24	17	5	64
		%	3.1	12.5	9.4	3.1	37.5	26.6	7.8	100.0
	Yes	*n*	3	3	1	1	7	9	7	31
		%	9.7	9.7	3.2	3.2	22.6	29.0	22.6	100.0
Need for lumbar puncture	No	*n*	2	3	1	2	9	2	4	23
		%	8.7	13.0	4.3	8.7	39.1	8.7	17.4	100.0
	Yes	*n*	3	8	6	1	22	24	8	72
		%	4.2	11.1	8.3	1.4	30.6	33.3	11.1	100.0
Need for electroencephalogram	No	*n*	4	10	6	3	28	22	11	84
		%	4.8	11.9	7.1	3.6	33.3	26.2	13.1	100.0
	Yes	*n*	1	1	1	0	3	4	1	11
		%	9.1	9.1	9.1	0.0	27.3	36.4	9.1	100.0
MRI findings	Abnormal	*n*	2	2	1	3	12	9	3	32
		%	6.3	6.3	3.1	9.4	37.5	28.1	9.4	100.0
	Normal	*n*	1	3	0	0	2	5	4	15
		%	6.7	20.0	0.0	0.0	13.3	33.3	26.7	100.0
	Not done	*n*	2	6	6	0	17	12	5	48
		%	4.2	12.5	12.5	0.0	35.4	25.0	10.4	100.0
Outcome	Death	*n*	0	0	3	0	0	0	1	4
		%	0.0	0.0	75.0	0.0	0.0	0.0	25.0	100.0
	Improving	*n*	2	5	4	3	20	12	7	53
		%	3.8	9.4	7.5	5.7	37.7	22.6	13.2	100.0
	Resolved	*n*	3	6	0	0	11	14	4	38
		%	7.9	15.8	0.0	0.0	28.9	36.8	10.5	100.0

The most common diagnosis in the recovered patient was meningoencephalitis (*n* = 20). All the patients who died during the treatment had associated comorbidities but no concurrent infections. Altered sensorium, fever, and headache were the common presenting symptoms in all the recovered patients. Five patients each in a resolved and improving group had their EEG done (Table 3).

**Table 3:** Outcome‐wise detailed characteristics of the study population (*n* = 95).

			Death (*n* = 4)	Improving (*n* = 53)	Resolved (*n* = 38)	Row total
Age			59.25 ± 25.51	40.58 ± 17.87	50.10 ± 19.85	
Duration of hospital stay			12.25 ± 12.00	12.96 ± 9.67	15.55 ± 12.18	
Sex	Female	*n*	1	23	15	39
		%	2.6	59.0	38.5	100.0
	Male	*n*	3	30	23	56
		%	5.4	53.6	41.1	100.0
Province number	1	*n*	1	1	0	2
		%	50.0	50.0	0.0	100.0
	2	*n*	0	6	7	13
		%	0.0	46.2	53.8	100.0
	3	*n*	1	27	18	46
		%	2.2	58.7	39.1	100.0
	4	*n*	2	13	9	24
		%	8.3	54.2	37.5	100.0
	5	*n*	0	6	1	7
		%	0.0	85.7	14.3	100.0
	7	*n*	0	0	3	3
		%	0.0	0.0	100.0	100.0
Etiology	Autoimmune	*n*	0	2	3	5
		%	0.0	40.0	60.0	100.0
	Bacterial	*n*	0	5	6	11
		%	0.0	45.5	54.5	100.0
	Fungal	*n*	3	4	0	7
		%	42.9	57.1	0.0	100.0
	Unknown	*n*	1	7	4	12
		%	8.3	58.3	33.3	100.0
	Tapeworm	*n*	0	3	0	3
		%	0.0	100.0	0.0	100.0
	Tubercular	*n*	0	20	11	31
		%	0.0	64.5	35.5	100.0
	Viral	*n*	0	12	14	26
		%	0.0	46.2	53.8	100.0
Diagnosis	Encephalitis	*n*	0	8	8	16
		%	0.0	50.0	50.0	100.0
	Meningitis	*n*	2	23	10	35
		%	5.7	65.7	28.6	100.0
	Meningoencephalitis	*n*	2	22	20	44
		%	4.5	50.0	45.5	100.0
Marital status	Married	*n*	3	40	35	78
		%	3.8	51.3	44.9	100.0
	Unmarried	*n*	1	13	3	17
		%	5.9	76.5	17.6	100.0
Comorbidities	Cardiac disease	*n*	1	8	6	15
		%	6.7	53.3	40.0	100.0
	Diabetes	*n*	2	4	3	9
		%	22.2	44.4	33.3	100.0
	None	*n*	0	32	19	51
		%	0.0	62.7	37.3	100.0
	Others	*n*	1	9	10	20
		%	5.0	45.0	50.0	100.0
Concurrent infections	Yes	*n*	0	16	5	21
		%	0.0	76.2	23.8	100.0
	No	*n*	4	37	33	74
		%	5.4	50.0	44.6	100.0
Previous treatment/referral	No	*n*	3	21	14	38
		%	7.9	55.3	36.8	100.0
	Yes	*n*	1	32	24	57
		%	1.8	56.1	42.1	100.0
History of surgery	No	*n*	4	45	36	85
		%	4.7	52.9	42.4	100.0
	Yes	*n*	0	8	2	10
		%	0.0	80.0	20.0	100.0
Invasive device placed	No	*n*	4	48	38	90
		%	4.4	53.3	42.2	100.0
	Yes	*n*	0	5	0	5
		%	0.0	100.0	0.0	100.0
Fever	No	*n*	3	14	14	31
		%	9.7	45.2	45.2	100.0
	Yes	*n*	1	39	24	64
		%	1.6	60.9	37.5	100.0
Headache	No	*n*	2	19	23	44
		%	4.5	43.2	52.3	100.0
	Yes	*n*	2	34	15	51
		%	3.9	66.7	29.4	100.0
Vomiting	No	*n*	4	32	25	61
		%	6.6	52.5	41.0	100.0
	Yes	*n*	0	21	13	34
		%	0.0	61.8	38.2	100.0
Altered sensorium	No	*n*	2	24	13	39
		%	5.1	61.5	33.3	100.0
	Yes	*n*	2	29	25	56
		%	3.6	51.8	44.6	100.0
Seizures	No	*n*	4	35	24	63
		%	6.3	55.6	38.1	100.0
	Yes	*n*	0	18	14	32
		%	0.0	56.3	43.8	100.0
Neck stiffness	No	*n*	2	33	25	60
		%	3.3	55.0	41.7	100.0
	Yes	*n*	2	20	13	35
		%	5.7	57.1	37.1	100.0
Kernig sign	No	*n*	2	39	32	73
		%	2.7	53.4	43.8	100.0
	Yes	*n*	2	14	6	22
		%	9.1	63.6	27.3	100.0
Brudzinski sign	No	*n*	4	45	31	80
		%	5.0	56.3	38.8	100.0
	Yes	*n*	0	8	7	15
		%	0.0	53.3	46.7	100.0
Papilledema	No	*n*	4	52	37	93
		%	4.3	55.9	39.8	100.0
	Yes	*n*	0	1	1	2
		%	0.0	50.0	50.0	100.0
Focal neurologic deficit	No	*n*	4	36	24	64
		%	6.3	56.3	37.5	100.0
	Yes	*n*	0	17	14	31
		%	0.0	54.8	45.2	100.0
Need for lumbar puncture	No	*n*	1	13	9	23
		%	4.3	56.5	39.1	100.0
	Yes	*n*	3	40	29	72
		%	4.2	55.6	40.3	100.0
Need for electroencephalogram	No	*n*	3	48	33	84
		%	3.6	57.1	39.3	100.0
	Yes	*n*	1	5	5	11
		%	9.1	45.5	45.5	100.0
MRI findings	Abnormal	*n*	1	14	17	32
		%	3.1	43.8	53.1	100.0
	Normal	*n*	1	8	6	15
		%	6.7	53.3	40.0	100.0

## 4 DISCUSSION

Infections involving the brain, spinal cord, optic nerves, and meninges‐collectively known as CNS infections are among the most debilitating conditions associated with significant morbidity, mortality, and lasting complications. Due to limited space and the presence of vital structures inside the brain, CNS infections are associated with high morbidity and mortality.^7^ To ensure optimum disease outcome, early diagnosis and prompt management is very crucial. For this, recognizing the local disease patterns in terms of disease distribution, commonly implicated aetiologies, presenting symptoms and signs, and prognostic factors is of utmost importance. An evidence‐driven management protocol catering to the local disease pattern serves as an invaluable guide to the clinicians in obtaining favorable disease outcomes. Despite the overwhelming need, the technical and economic constraints hinder conducting studies that reflect the epidemiology of CNS infections, especially in a limited resource setting like Nepal. Our study aimed to bridge this gap somehow by outlining the epidemiological profile of CNS infections in a tertiary health care center in Nepal with respect to age, etiology, and the outcome of the disease.

Acute CNS infections can be broadly categorized into meningitis, encephalitis, and abscesses.^8^ Meningoencephalitis was the most commonly diagnosed CNS infection in the center where our study was based, followed by meningitis and encephalitis. The clinical presentation of meningitis was heterogeneous with patients very rarely presenting with the classical triad of fever, nuchal rigidity, and meningismus.^9^ Fever (67.40%, *n* = 64, *N* = 95), altered sensorium (58.90%, *n* = 56, *N* = 95), and headache (53.60%, *n* = 51, *N* = 95) were the most common presenting symptoms. 32.60% (*n* = 31, *N* = 95) of the study population presented with some forms of focal neurological deficit as well. On examination, only 36.80% (*n* = 35, *N* = 95) patients showed neck stiffness. Our results correspond with a similar study done in India that studied the clinical spectrum of CNS infections which also highlighted fever (94.5%) as the most common presenting symptom followed by altered mental status (76.6%) and headache (70.6%).^10^

The most encountered age group was 30−60 years, although no deaths occurred in this age group. The number of male patients in every age group was higher except in the 10−30 age range where 17 out of the total 27 patients were females. A multinational study outlining the burden of CNS infections found that the mean age of cases was 47.63 years which does not stray too far from our observation.^11^ Tubercular (32.60%) was the most common etiology, followed by viral (27.40%). This finding is consistent with another observational study conducted in Malaysia where tuberculous meningitis was not only the most diagnosed CNS infection, but also a cause of significant mortality and morbidity among the patients.^5^ The male: female ratio in our study was 1.43:1 which is in line with the finding of a study done in China^12^ (3:1) but in contrast to the global data obtained from a comprehensive meta‐analysis (1:1.74).^1^ Almost half of the patients were from Province 3 (Bagmati), and the location of our institution in the same province explains this data. Fungal, although comprising only 7.40% (*n* = 7, *N* = 95) of the total CNS infections, account for 75% (*n* = 3, *N* = 4) of total deaths. All the deaths were in those who had at least one form of preexisting comorbidities.

Diagnosis is made considering multiple parameters (but not all) which includes clinical features, imaging findings, CSF analysis, nucleic acid amplification test, and in some cases of epidemiological aspects of the disease too. CSF analysis is performed in most of the patients. Definite etiological diagnosis is sometimes not possible within the country because of infrastructure and technology limitations. In such cases we send samples to foreign country for analysis depending on the patient's affordability. Lumbar puncture is a vital tool in the diagnosis and management of CNS infections. It tailors the treatment toward bacterial, viral, tubercular, fungal, or noninfectious diagnosis.^13^ Although lumbar puncture is one of the most commonly used investigations to diagnose CNS infections, our study found that lumbar puncture was performed in only 75.80% (*n* = 72, *N* = 95) of the cases. Our hospital is a major referral center of the country. Some of the already diagnosed cases of CNS infections are referred for further management and repetition of lumbar puncture is such cases are avoided in our center. Neuroimaging especially MRI, can allow the identification of infection pattern, differentiating them from vascular disease and neoplasm, and even signal toward the causative agent.^14^ Because of its time‐consuming nature, and infrastructural and maintenance limitations in developing countries like Nepal, MRI is not widely available and utilized in the diagnosis of CNS infections. Half of the patients in our study didn't have their MRI done, and 33.79% (*n* = 32, *N* = 95) had abnormal MRI findings. The most common etiology in the age group of 10−30 years was also tubercular (12 out of 27 patients) followed by viral, bacterial, and autoimmune in decreasing order. According to a study done in Vietnam, the most commonly implicated organism of CNS infections in patients under the age of 14 was JEV, which was not the case in our study.^15^ Interestingly, the number of CNS viral infections were equal to tubercular in the 30−60 age group.

The age‐disaggregated data of our study shows that 75% (*n* = 3, *N* = 4) of deaths occurred in the age group of 60−90. This occurrence may be explained by a higher rate of preexisting comorbidities in this age group. Tubercular CNS infection was the most common diagnosis (32.60%, *n* = 31, *N* = 95) which is unsurprising as the TB prevalence rates in Nepal are still high. Viral etiology comes second (27.40%, *n* = 26, *N* = 95). These findings are incongruent with a similar study done in another tertiary center of Nepal which found that viral CNS etiologies were the most common.^3^ Out of our study population of 95, 44 have comorbidities including but not limited to diabetes mellitus and cardiovascular diseases. We also found out that the prognosis of CNS infections was worse among the patients with comorbidities, which may have to do with a relatively immunocompromised state in this cohort.^16^

Treatment of CNS infections depends on the type of causative organism. For pyogenic infection we start with empirical antibiotics (ceftriaxone and vancomycin), later choice of antibiotic is guided by culture and sensitivity report of the culture. In case of immunocompromised patient, we add ampicillin and aminoglycosides. For fungal infection like cryptococcal we initiate therapy with fluconazole and amphotericin B according to standard protocol. For Herpes simplex infections, acyclovir therapy is initiated and symptomatic treatment is used for other viral infections. Antitubercular regimen for tubercular infection in our center is 2 months of isoniazid, rifampicin, pyrazinamide, and ethambutol followed by 10 months of isoniazid, rifampicin, and ethambutol along with steroids.

Our study had a few limitations that need to be mentioned here. Because of the retrospective nature of the study and improper maintenance of medical records, sample size for our study came out to be low. Detailed data in pathogen isolated, diagnosis and management were incomplete and missing, hence not included in the study. It is a single‐center study, and doesn't represent the findings of the whole country. Further large‐scale prospective studies are necessary to understand the burden, and epidemiology of CNS infections in Nepal. Nepal falls under the high burden region for CNS infections. However, there has been no study in Nepal that dissects the epidemiological and clinical characteristics of CNS infections. To our knowledge, this is the first comprehensive article with detailed findings of epidemiological and clinical characteristics of patients with diagnosis of CNS infections from Nepal which is one of our major strengths.

## 5 CONCLUSION

Our attempt to explore the epidemiological and clinical characteristics of CNS infections will be a credible source of information for academicians, clinicians, and an outset for future researchers. It can guide diagnosis and management of CNS infections.

## AUTHOR CONTRIBUTIONS

**Bikram P. Gajurel**: conceptualization; methodology; writing – review & editing. **Subarna Giri**: conceptualization; data curation; formal analysis; writing – original draft. **Shivani Rayamajhi**: data curation; writing – original draft. **Niharika Khanal**: data curation; writing – original draft. **Sagar Bishowkarma**: data curation; writing – original draft. **Aman Mishra**: methodology; writing – review & editing. **Ragesh Karn**: supervision. **Reema Rajbhandari**: supervision. **Rajeev Ojha**: supervision.

## CONFLICT OF INTEREST STATEMENT

The authors declare no conflict of interest.

## TRANSPARENCY STATEMENT

The lead author Subarna Giri affirms that this manuscript is an honest, accurate, and transparent account of the study being reported; that no important aspects of the study have been omitted; and that any discrepancies from the study as planned (and, if relevant, registered) have been explained.

## Data Availability

The data that support the findings of the present study are available on reasonable request. However, they are not publicly available due to privacy and ethical restrictions.
